# Supplementary material for: The DNA virome varies with human genes and environments
Source: Nature. 2026 Mar 25;653(8116):1099–109. doi: 10.1038/s41586-026-10288-y (PMC13215884; doi:10.1038/s41586-026-10288-y)
Supplement: Supplementary file 2 — Reporting Summary [file 41586_2026_10288_MOESM2_ESM.pdf]

nature portfolio

Corresponding author(s): Nolan Kamitaki

Last updated by author(s): Feb 11, 2026

Reporting Summary

Nature Portfolio wishes to improve the reproducibility of the work that we publish. This form provides structure for consistency and transparency in reporting. For further information on Nature Portfolio policies, see our Editorial Policies and the Editorial Policy Checklist.

Statistics

For all statistical analyses, confirm that the following items are present in the figure legend, table legend, main text, or Methods section.

n/a	Confirmed	
☐	☒	The exact sample size (*n*) for each experimental group/condition, given as a discrete number and unit of measurement
☐	☒	A statement on whether measurements were taken from distinct samples or whether the same sample was measured repeatedly
☐	☒	The statistical test(s) used AND whether they are one- or two-sided<br>*Only common tests should be described solely by name; describe more complex techniques in the Methods section.*
☐	☒	A description of all covariates tested
☐	☒	A description of any assumptions or corrections, such as tests of normality and adjustment for multiple comparisons
☐	☒	A full description of the statistical parameters including central tendency (e.g. means) or other basic estimates (e.g. regression coefficient) AND variation (e.g. standard deviation) or associated estimates of uncertainty (e.g. confidence intervals)
☐	☒	For null hypothesis testing, the test statistic (e.g. *F*, *t*, *r*) with confidence intervals, effect sizes, degrees of freedom and *P* value noted<br>*Give P values as exact values whenever suitable.*
☒	☐	For Bayesian analysis, information on the choice of priors and Markov chain Monte Carlo settings
☒	☐	For hierarchical and complex designs, identification of the appropriate level for tests and full reporting of outcomes
☐	☒	Estimates of effect sizes (e.g. Cohen's *d*, Pearson's *r*), indicating how they were calculated

Our web collection on statistics for biologists contains articles on many of the points above.

Software and code

Policy information about availability of computer code

Data collection	No software was used for data collection.
Data analysis	The following publicly available software resources were used: bwa (v0.7.18, https://bio-bwa.sourceforge.net/), mosdepth (v0.3.9, https://github.com/brentp/mosdepth), bcftools (v1.14, http://www.htslib.org/), samtools (v1.15.1, http://www.htslib.org/), plink (v1.90b6.26 and v2.00a3.7, https://www.cog-genomics.org/plink/), BEAGLE (v5.4, https://faculty.washington.edu/browning/beagle/beagle.html), BOLT-LMM (v2.5, https://alkesgroup.broadinstitute.org/BOLT-LMM/), METAL (v2020-05-05, https://genome.sph.umich.edu/wiki/METAL), qqman R package (v0.1.8, https://cran.r-project.org/web/packages/qqman/index.html), sf R package (v1.0-20, https://cran.r-project.org/web/packages/sf/index.html), logistf R package (v1.26.1, https://cran.r-project.org/web/packages/logistf/index.html), and MendelianRandomization R package (v0.10.0, https://cran.r-project.org/web/packages/MendelianRandomization/index.html).

For manuscripts utilizing custom algorithms or software that are central to the research but not yet described in published literature, software must be made available to editors and reviewers. We strongly encourage code deposition in a community repository (e.g. GitHub). See the Nature Portfolio guidelines for submitting code & software for further information.

Data

Policy information about availability of data

All manuscripts must include a data availability statement. This statement should provide the following information, where applicable:

- Accession codes, unique identifiers, or web links for publicly available datasets
- A description of any restrictions on data availability
- For clinical datasets or third party data, please ensure that the statement adheres to our policy

The following data resources are available by application: UK Biobank (http://www.ukbiobank.ac.uk/), All of Us Research Program (https://allofus.nih.gov/), SFARI SPARK (https://www.sfari.org/resource/spark/), MVP-Finngen-UKBB meta-analysis summary statistics (https://mvp-ukbb.finngen.fi/), and T1DGC HLA imputation panel (https://repository.niddk.nih.gov/study/173). The following data resources are publicly available: human reference genome build GRCh38 (https://ftp.1000genomes.ebi.ac.uk/vol1/ftp/technical/reference/GRCh38_reference_genome/), TOPMed-r2 imputation panel variant list (https://imputation.biodatacatalyst.nhlbi.nih.gov/), gnomAD v4.1 variant call set (https://gnomad.broadinstitute.org/), LD score resources https://alkesgroup.broadinstitute.org/LDSCORE/), NCBI Virus for reference sequences (https://www.ncbi.nlm.nih.gov/labs/virus/vssi/), PrimateAI-3D scores (https://primateai3d.basespace.illumina.com/), GENCODE 39 definitions (https://www.gencodegenes.org/), and GTEx expression and splice quantitative trait associations (https://gtexportal.org/home/). Full viral DNA load GWAS summary statistics are available from the GWAS Catalog under accessions GCST90809801 to GCST90809829.

Research involving human participants, their data, or biological material

Policy information about studies with human participants or human data. See also policy information about sex, gender (identity/presentation), and sexual orientation and race, ethnicity and racism.

Reporting on sex and gender	For UK Biobank (recorded 222,094 males, 263,132 females), sex was acquired from NHS central registry at recruitment, but in some cases updated by the participant. For All of Us (recorded 161,253 males, 252,074 females), sex was genetically determined by copy number and presence of X and Y chromosomes. For SFARI SPARK (recorded 7284 males, 5235 females), sex was self-reported. Sex was used as a covariate in most analyses (ex. human genetic associations with viral load), where no values directly pertaining to sex are reported for these analyses. Values pertaining to sex were reported for analyses that demonstrated higher viral load in men (Fig. 2a-d, EDF 3c-e).
Reporting on race, ethnicity, or other socially relevant groupings	UK Biobank, using the top 20 ancestry principal components, a subset of individuals that fell within a Euclidean distance (centered at the mean values of each PC for individuals who self-identified as "white") capturing 99% of individuals who self-identified as "white" were used for genotype and phenotype associations. For All of Us, analyses were restricted to individuals with previously released genetically-predicted European ancestry for genotype and phenotype associations. For SPARK, using the top 10 ancestry principal components, a subset of individuals that fell within a Euclidean distance (centered at the mean values of each PC for individuals who self-identified as "white") capturing 90% of individuals who self-identified as "white" were used for genotype and phenotype associations. For all analyses, ancestry principal components were included as covariates in genetic associations. For associations of viral DNA with ancestry, these genetically-predicted ancestries were used as independent variables for association with viral DNA.
Population characteristics	UK Biobank is a cohort of approximately 500,000 individuals across the United Kingdom between 40 and 69 years of age at time of recruitment (Sudlow et al. 2015 PLOS Medicine). For viral DNA associations in the UK Biobank cohort, age, age squared, sex, genotype array, assessment center, and top 20 genetic ancestry PCs were used as covariates. All of Us is a cohort of 414,817 individuals with WGS available (at time of analysis) across the United States older than 18 years of age at time of recruitment (The All of Us Research Program Investigators 2019 N Engl J Med). For viral DNA associations in the All of Us cohort, age, age squared, sex, and the top 16 genetic ancestry principal components (from ancestry_preds.tsv) were used as covariates. SFARI SPARK is a cohort of approximately 160,000 families with at least one child with autism spectrum disorder, where 12,519 individuals (at time of analysis) have WGS from saliva available (SPARK Consortium 2018 Neuron). Children range in age from 0-50 years of age (mean 9 years) and parents from 19-90 years of age (mean 41 years). For viral DNA associations in the SPARK SFARI cohort, sequencing batch, age, age squared, square root of age, sex, percent of mapped reads, and the top 10 genetic ancestry principal components were used as covariates.
Recruitment	Individuals and biosamples were not obtained for this study and their recruitment is as described in prior publications (cited in current work).
Ethics oversight	Individuals and biosamples were not obtained for this study and local IRBs at each institution approved the collections and patient-consent materials, as described in the earlier papers on these cohorts (cited in current work). Datasets were used as approved for research plans as stated in applications to each, including UK Biobank Resource application #40709.

Note that full information on the approval of the study protocol must also be provided in the manuscript.

Field-specific reporting

Please select the one below that is the best fit for your research. If you are not sure, read the appropriate sections before making your selection.

☒ Life sciences ☐ Behavioural & social sciences ☐ Ecological, evolutionary & environmental sciences

For a reference copy of the document with all sections, see nature.com/documents/nr-reporting-summary-flat.pdf

Life sciences study design

All studies must disclose on these points even when the disclosure is negative.

Sample size	For associations in the UK Biobank WGS data set, individuals were excluded based on the following criteria: removed for not having European genetic ancestry; removed for not having available TOPMed-imputed genotypes (including for chromosome X); and removed for having withdrawn, leaving 453,770 available individuals for genetic association analyses. For some associations using linear regression rather than linear mixed models that would account for relatedness (ex. HLA local associations), additional samples were removed to drop one relative within pairs of close relatives with kinship coefficient > 0.0884.<br><br>For associations in the All of Us cohort, 414,817 samples with available WGS were first separated into those where sample DNA was either blood (n=365,918) or saliva-derived (n=48,899). Individuals were then excluded for not having European genetic ancestry, leaving 201,181 blood-derived samples and 33,164 saliva-derived samples. For analyses of HHV-6B viral load, individuals with endogenous HHV-6A or HHV-6B were excluded, leaving 199,133 blood-derived samples and 32,826 saliva samples. For saliva-derived samples, some associations (such as those associating quantity rather than presence of viral DNA) were then performed on the subset of samples with at least 1 viral DNA sequence, leaving a subset of samples as noted in the text (ex. 16,282 samples for EBV).<br><br>For associations in the SFARI SPARK cohort, among 12,519 samples with available WGS data some samples were removed for not having European genetic ancestry, leaving 9,209 individuals. For analyses of HHV-6B viral load, individuals with endogenous HHV-6A or HHV-6B were excluded, leaving 9,081 individuals. Some associations associating quantity rather than presence of viral DNA were then performed on the subset of samples with at least 1 viral DNA sequence, leaving a reduced sample number as noted in the text.<br><br>In all cases, no sample-size calculation was done to predetermine sample size and the maximum number of available samples were used. For viral DNA genetic associations, we expected that although the phenotype was sparser with less dynamic range than previous work measuring viral load by PCR (ex. HIV viral load), that the greatly increased sample size (2-3 orders of magnitude) would allow for observing expected associations in the MHC region of the human genome. Previous work has also suggested EBV viral load to be heritable.
Data exclusions	Established QC metrics were used to exclude some samples, genotypes, or sequencing data for analysis as described in previously published studies (cited in the current work). In brief: For UK Biobank WGS, the sequencing of 914 participants failed due to either insufficient or poor-quality DNA. For UK Biobank array genotypes used for imputation, ~3% of samples had insufficient DNA to use as input for genotyping and 968 samples with genotypes were excluded for high heterozygosity or >5% missing rate. For All of Us WGS, samples were excluded for low quality or insufficient DNA for library production and 987 samples were at least eight median absolute deviations from median residual in at least one type of variant metric (ex. number of SNPs, number of indels). For SPARK WGS, no samples were excluded by QC criteria. Samples from individuals in UK Biobank, All of Us, and SFARI SPARK that requested to be withdrawn at the time of analysis were excluded.
Replication	For each viral association observed in UK Biobank (blood) and SPARK (saliva), we were generally successful in replicating those tested in the subset of All of Us samples with DNA derived from the same source (blood or saliva) such that each had a single attempt at replication. This included associations with human genetics (besides one EBV-associated locus that had discordant direction of effect in blood samples from All of Us relative to UK Biobank), smoking, age, sex, time of day, and month of the year. We did not attempt replication of binary ICD-10 code phenotypes in All of Us.<br><br>Additionally, we attempted to verify all genetic associations to EBV and HHV-7 load in UK Biobank by generating additional phenotypes from reads aligning to the left and right halves of each genome and evaluating whether associations to all virally-aligned reads replicated in these phenotypes. All loci but 1 successfully replicated (the same locus which failed replication in All of Us), such that each association had a single attempt at replication with this approach.
Randomization	For UK Biobank, samples were collected in batches at different assessment centers at locations across the United Kingdom and these were encoded as indicator covariates in phenotype-genotype associations. For All of Us, samples were collected in batches at different sequencing centers, and these were encoded as indicator variables in phenotype-genotype associations. For SFARI SPARK, samples were collected in different batches of sequencing cohorts (WGS1 through WGS5) and these were encoded as indicator covariates in phenotype-genotype associations. No further randomization was done in each cohort as all samples were used for each analysis.
Blinding	For data collection, blinding was not relevant as data was collected by other research groups (cited in the current work) and re-analyzed in this work. For all computational analyses blinding was always done, as samples were listed with a randomized ID where association of measured genotype with viral DNA phenotype was only done at the point of final statistical analysis.

Reporting for specific materials, systems and methods

We require information from authors about some types of materials, experimental systems and methods used in many studies. Here, indicate whether each material, system or method listed is relevant to your study. If you are not sure if a list item applies to your research, read the appropriate section before selecting a response.

Materials & experimental systems

n/a	Involved in the study
☒	☐ Antibodies
☒	☐ Eukaryotic cell lines
☒	☐ Palaeontology and archaeology
☒	☐ Animals and other organisms
☒	☐ Clinical data
☒	☐ Dual use research of concern
☒	☐ Plants

Methods

n/a	Involved in the study
☒	☐ ChIP-seq
☒	☐ Flow cytometry
☒	☐ MRI-based neuroimaging

Plants

Seed stocks

Report on the source of all seed stocks or other plant material used. If applicable, state the seed stock centre and catalogue number. If plant specimens were collected from the field, describe the collection location, date and sampling procedures.

Novel plant genotypes

Describe the methods by which all novel plant genotypes were produced. This includes those generated by transgenic approaches, gene editing, chemical/radiation-based mutagenesis and hybridization. For transgenic lines, describe the transformation method, the number of independent lines analyzed and the generation upon which experiments were performed. For gene-edited lines, describe the editor used, the endogenous sequence targeted for editing, the targeting guide RNA sequence (if applicable) and how the editor was applied.

Authentication

Describe any authentication procedures for each seed stock used or novel genotype generated. Describe any experiments used to assess the effect of a mutation and, where applicable, how potential secondary effects (e.g. second site T-DNA insertions, mosiacism, off-target gene editing) were examined.
